# Case-control study: Unveiling human polyomaviruses and papillomavirus in Egyptian colorectal cancer patients

**DOI:** 10.1371/journal.pone.0304147

**Published:** 2024-06-11

**Authors:** Marwa K. Darwish, Abdou K. Allayeh, Amr E. Ahmed, Mohamed D. E. Abdelmaksoud, Samia S. Alkhalil, Mohammed Ageeli Hakami, Ahmed Hassan, Haiam Mohamed Mahmoud Farrag, Samah Saif Eldin M. Mohamed, Weaam Gouda

**Affiliations:** 1 Department of Clinical Laboratory Sciences, College of Applied Medical Sciences, Shaqra University, Al-Quwayiyah, Riyadh, Saudi Arabia; 2 Chemistry Department (Biochemistry Branch), Faculty of Science, Suez University, Suez, Egypt; 3 Virology Lab, Water Pollution Research Department, Environment and Climate Change Institute, National Research Centre, Giza, Egypt; 4 Department of Biotechnology and Life Science, Faculty of Postgraduate Studies for Advanced Sciences, Beni-Suef University, Beni‑Suef, Egypt; 5 Biochemistry Department, Biotechnology Research Institute, National Research Centre, Giza, Egypt; 6 Oncology Department, Faculty of Medicine, Beni-Suef University, Beni‑Suef, Egypt; University of Nebraska-Lincoln, UNITED STATES

## Abstract

**Background:**

Colorectal cancer (CRC) is a cancer type that is thought to be influenced by human papillomaviruses (HPVs) and human polyomaviruses (HPyVs). In Egypt, CRC ranks as the 7th most common cancer, accounting for 3.47% of male cancers and 3% of female cancers. However, there is currently a lack of information regarding the presence of PyVs and HPVs co-infection specifically in CRC cases in Egypt. Therefore, the aim of this study was to investigate the occurrence of HPVs and HPyVs (JCPyV, BKPyV, and SV40) infections, as well as co-infections, among CRC patients in Egypt. Additionally, the study aimed to assess any potential association between these viral infections and tumor stages.

**Methods:**

In the present study, we analyzed a total of 51 tissue samples obtained from Egyptian CRC patients, along with 19 polyps’ samples. Our investigation focused on the detection and genotyping of HPyVs using Real-Time PCR. Additionally, we employed real-time PCR for the detection of HPVs, and for their genotyping, we utilized a combination of PCR amplification followed by sequencing.

**Results:**

In our study, we found evidence of HPyVs infection in the CRC patients, specifically SV40 (25.5%) and BKPyV (19.6%). However, JCPyV was not detected in the samples that were examined. Additionally, we discovered that HPV was present in 43.1% of the CRC patients. When considering viral co-infections, 19.6% of the CRC samples showed coexistence of multiple viruses, while no co-infections were found in the polyps samples. Importantly, we observed a significant correlation between the presence of HPVs and advanced colorectal tumor grades B2 and D.

**Conclusion:**

Our findings provide valuable data for the detection of oncogenic viruses in colorectal cancer (CRC) and underscore the association of viral co-infections with advanced tumor stages. However, further research with larger cohorts is necessary to validate these findings and strengthen their significance in the field of CRC.

## Introduction

Colorectal cancer (CRC) is the second most prevalent cause of death due to cancer globally, accounting for 10% of the total deaths related to cancer [1]. Furthermore, according to Kim et al., CRC contributes to 13% of all gastrointestinal tract malignancies [2] and is the second most frequent cancer type in women and the third most common in men [3]. The incidence of CRC is higher in people older than 50 years old, and symptoms that are most common include anemia, hematochezia, losing weight, abdominal pains, and alterations in gastrointestinal habits [4]. Multiple risk factors, especially genetic, hereditary, environmental, and infectious agents, have been linked to the etiology of colorectal cancer [5]. Colorectal cancers often originate from polyps, which are growths on the inner lining of the colon or rectum. While not all polyps become cancerous, certain types, such as adenomatous polyps (adenomas), have the potential to transform into cancer over time, making them a pre-cancerous condition. Adenomas can be categorized as tubular, villous, or tubulovillous. Hyperplastic polyps and inflammatory polyps are more common but generally not pre-cancerous, although larger hyperplastic polyps may necessitate more frequent colorectal cancer screening. Sessile serrated polyps (SSP) and traditional serrated adenomas (TSA) pose a higher risk of colorectal cancer and are treated similarly to adenomas. Factors that increase the likelihood of cancer within polyps or the overall risk of developing colorectal cancer include larger polyp size, the presence of multiple polyps, and the identification of dysplasia, which indicates abnormal cells that have not yet become cancerous [6]. In 1932, British pathologist Cuthbert Dukes (1890–1977) created a classification system for colorectal cancer. This system, known as the Dukes classification, included several variations. However, it has largely been supplanted by the more comprehensive TNM staging system and is no longer recommended for clinical use. The Dukes classification consisted of the following stages: Dukes A, which indicated invasion into the bowel wall but not through it; Dukes B, signifying invasion through the bowel wall, penetrating the muscle layer but without involvement of lymph nodes; Dukes C, denoting the presence of lymph node involvement; and Dukes D, representing widespread metastases [7].

Owing to the enormous diversity of microbes in the gastrointestinal tract of humans and the detection of infectious agents as potential contributors to various types of cancer, investigations worldwide showed a correlation amongst several infectious pathogens and the development of colorectal neoplasm [8]. The World Health Organization has identified various viral, bacterial, and parasitic pathogens as human carcinogens such as *Helicobacter pylori*, *Fusobacterium nucleatum*, *Bacteroides fragilis*, *Streptococcus gallolyticus*, *Escherichia coli*, *Schistosoma japonicum*, *and Blastocystis*. Infections are estimated to contribute to approximately 15% of cancer cases worldwide, making them a significant modifiable risk factor for cancer. While no specific microbial pathogens have been definitively linked to colorectal cancer (CRC) to date, emerging evidence suggests that dysbiosis, can promote carcinogenesis in humans. This can occur through chronic inflammation, immune subversion, and the production of carcinogenic metabolites. Among viral pathogens with oncogenic potential, different human polyomaviruses (HPyVs) and human papillomaviruses (HPVs) have been investigated for their association with human cancers [9–12].

Polyomaviruses, a family of non-enveloped viruses, exhibit icosahedral capsids that enclose small circular genomes consisting of double-stranded DNA [13]. Within this family, human SV40, BKPyV, and JCPyV are the most common viruses. These particular non-enveloped viruses possess circular genomes spanning approximately 5000 base pairs. While the majority of polyomaviruses do not infect humans, there have been 14 polyomaviruses identified with human hosts as of 2017. Nonetheless, specific polyomaviruses are associated with human diseases, particularly among individuals with compromised immune system and those afflicted with cancers [13]. Several human polyomaviruses, including BK virus (BKPyV) and JC virus (JCPyV), were isolated from immunocompromised patients in 1971 and are members of the *polyomaviridae* family [14]. Millions of individuals worldwide were unintentionally exposed to a third polyomavirus, Simian virus 40 (SV40), in the late 1950s and early 1960s due to administration of contaminated polio vaccines [15]. The polyomaviruses BKPyV, JCPyV, and SV40 are being investigated more closely as potential cofactors in human cancer. They have also been linked to a number of human disorders [12].

The most studied human polyomaviruses are JC (JCPyV) and BK (BKPyV) polyomaviruses, adult sero-prevalence for BKPyV and JCPyV is very high. While the initial BKPyV and JCPyV infection appears to be asymptomatic and maintains the host with a harmless latent infection throughout life, reactivation of the virus in immune compromised individuals might cause disease [12]. It is speculated that JCPyV is an opportunistic pathogen that infects children during their first few years of life [16]. Adults are susceptible to JCPyV, with a prevalence of 50 to 60 percent [17] and the prevalence extents 70% in the elderly [18]. The potential significance of JCPyV in the development of gastrointestinal malignancies, including as CRC, has been studied [19, 20]. It is yet unclear whether JCPyV and the development of CRC are causally related [10].

BK polyomavirus infection, which is prevalent in humans and mostly occurs in childhood, is defined as a tumor virus because of its tumorigenic effects in animal models as well as *in vitro* models of infections [21]. For example, it can immortalize human cells either by itself or in conjunction with other oncogene families, and it can turn rodent cells into tumors [22]. The mechanistic impact of BKPyV in human malignancies is still up for debate despite an enormous number of investigations. BKPyV has been detected in cancerous tissues from the bladder, prostate, pancreas, adrenal glands, and brain [23–26]. In contrast, in a number of research studies, BKPyV has not been found in comparable tumors [27–29].

Simian virus 40 (SV40) is another virus belongs to the *polyomaviridae* family. Up to 15% of the worldwide population possesses antibodies against simian virus 40, according to sero-epidemiological research, which supports the hypothesis that SV40 can infect humans horizontally and vertically [30]. SV40 belongs to the Betapolyomavirus genus, which is closely linked to human BKPyV and JCPyV [31]. Thus, SV40 and JCPyV and BKPyV have genomes that are approximately 70–75% similar [32]. The transforming and tumorigenic features of SV40 have been described in prior investigations and have been demonstrated in animal models and cell cultures [32, 33].

The genome of human papillomaviruses (HPVs) is approximately 8 kb in size [34]. It has been observed that HPVs can infect the colon and rectum [35]. HPVs represents a group of over 200 closely related viruses, some of which are transmitted through vaginal, anal, or oral sexual activities. Sexually transmitted HPV types are categorized into low-risk and high-risk groups. HPV is responsible for an estimated 630,000 cancer cases worldwide annually, accounting for approximately 5% of all cancers according to the World Health Organization [9]. According to Della Fera *et al*. persistent HPVs infection is thought to be crucial for the consequences of HPV-mediated malignancies, raising the probability of getting colorectal cancer [36]. Despite this, there aren’t plenty of investigations from the Middle East and North Africa region that have provided information on CRC related to HPVs. More so, the correlation between HPV infection and colorectal cancer remains controversial and inconclusive [8]. Finally, kazlauskas et al. [37] reported that eukaryotic human polyomaviruses and papillomaviruses, which possess double-stranded DNA genomes, have evolved from CRESS-DNA viruses, indicating a complex evolutionary history of this significant virus class and revealing its polyphyletic origins. This finding shed light on the intricate evolutionary pathways of these groups of viruses. Here, the present work aimed to assess the incidence of human papillomavirus (HPVs) and human polyomaviruses (JCPyV, BKPyV, and SV40) in colorectal cancer patients and to detect their viral co-infections in CRC sample tissues. Hence, this study aims to provide updated information on the incidence of oncogenic viruses, specifically human papilloma- and polyoma-viruses, as well as co-infections involving different types of these viruses.

## Results

Tables 1 and 2 in this study presented the demographic and incidence data, comparing the presence of HPyVs (Human Polyomaviruses) and HPVs (Human Papillomaviruses) in colorectal cancer (CRC) tissues (n = 51) with the control group of polyps (n = 19). Our analysis revealed that 45.1% (23/51) of the CRC samples tested positive for HPyVs, whereas only 3.2% of the polyps group showed HPyV presence. Among the HPyVs detected, SV40 was the most frequently found, present in 25.5% of the CRC group (p-value of 0.175) compared to 10.5% in the polyps group. BKPyV was detected in 19.6% (10/51) of the CRC group (p-value of 0.897) and in 21.1% (4/19) of the polyps group. However, JCPyV was not detected in any of the examined samples.

**Table 1:** Colon cancer patients and polyps groups’ demographic data.

		Colorectal Cancer Group (n = 51)	Polyps Group (n = 19)	*P*-Value
Age in years		60.24±9.8	53.86±8.3	**0.012**
Gender	Male	30 (58.8%)	13 (68.4%)	0.463
	Female	21 (41.2%)	6 (31.6%)	

Variables are presented as mean ± SD or as number of positive samples (%)

P value <0.05 are represented in bold font and considered as statistically significant

**Table 2:** Comparison between colon cancer patients and polyps groups’ viral incidence data.

	Colorectal Cancer Group (n = 51)	Polyps Group (n = 19)	*P*-Value
Human polyomaviruses (HPyVs)	23 (45.1%)	6 (3.2%)	
SV40	13 (25.5%)	2 (10.5%)	0.175
BKPyV	10 (19.6%)	4 (21.1%)	0.893
JCPyV	0 (0%)	0 (0%)	-
Human papillomaviruses (HPVs)	22 (43.1%)	5 (26.3%)	0.199
Co-infections	10 (19.6%)	0 (0%)	**0.037**

P value for comparison between colorectal cancer patients and polyps control groups

P value <0.05 are represented in bold font and considered as statistically significant

Additionally, HPVs were detected in 43.1% (22/51) of the CRC samples (p-value of 0.199) and in 26.3% (5/19) of the polyps group. Notably, co-infections of viruses were statistically significant (p = 0.037) in 19.6% (10/51) of the CRC samples, while no co-infections were detected in the polyps samples, as shown in Table 2.

**Fig 1:**
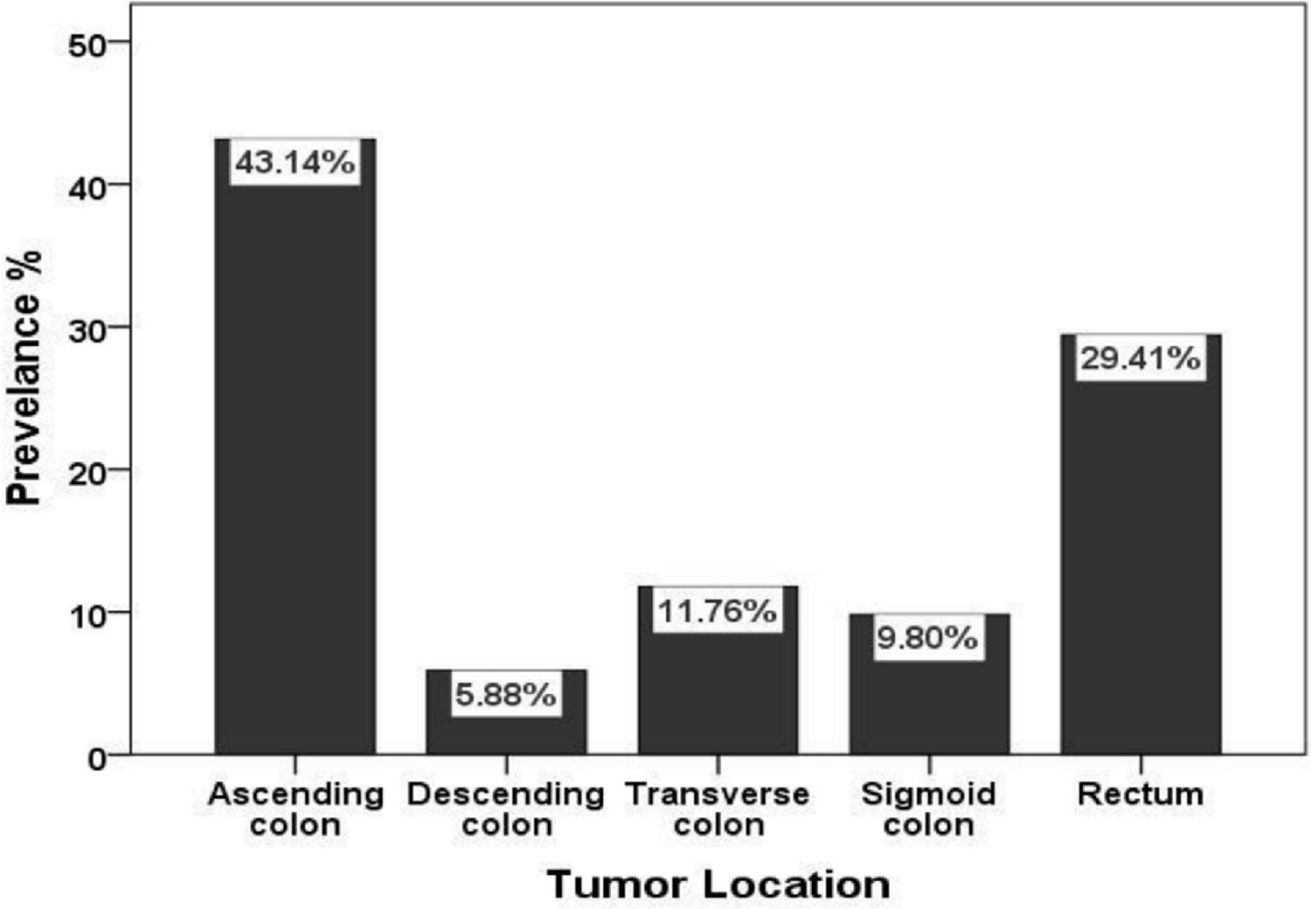
Tumor location in colorectal cancer patients.

**Fig 2:**
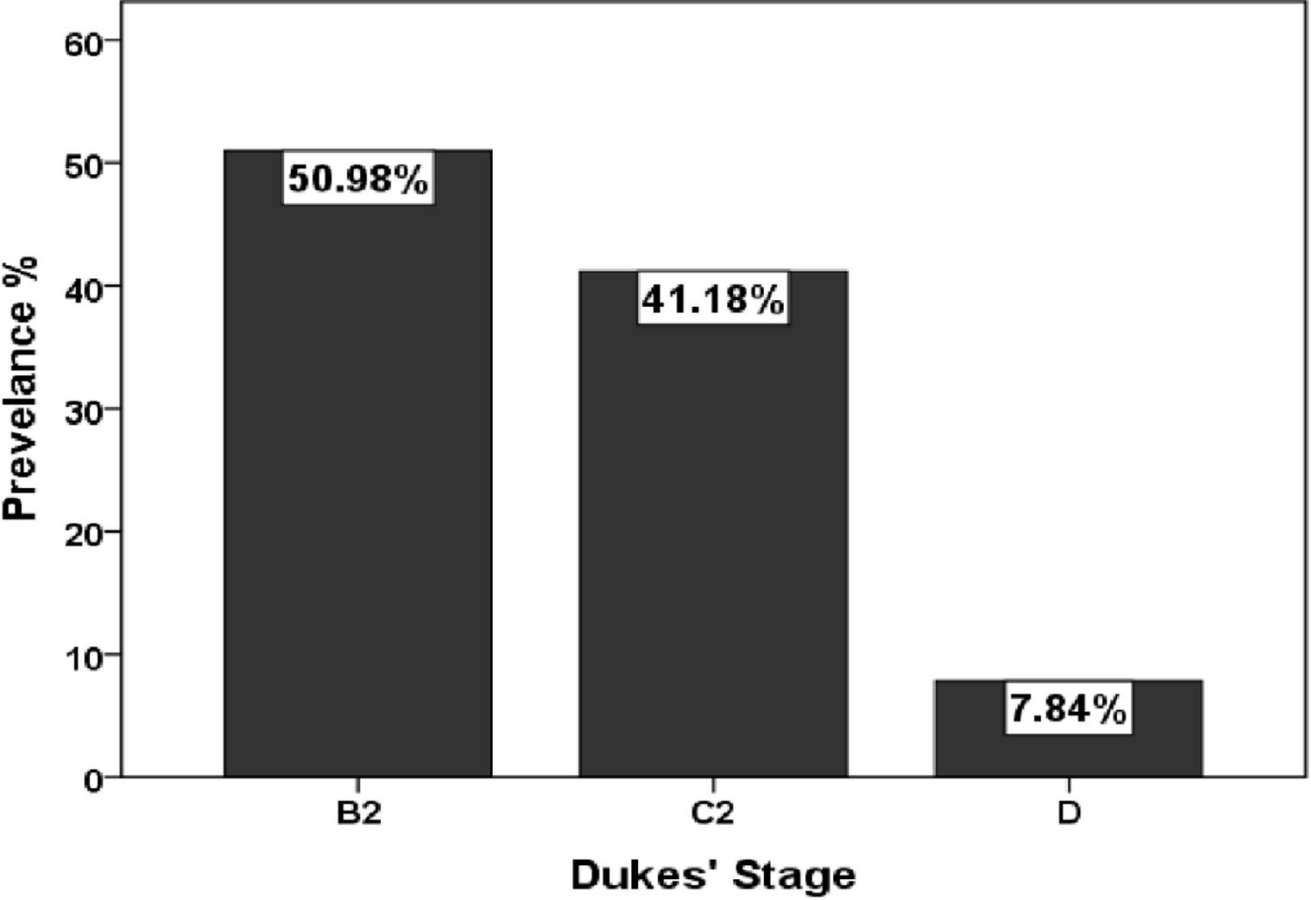
Tumor grades according to Dukes’ stage in patients with colorectal cancer.

As displayed in Fig 1, largest percentage of samples were collected from the ascending colon (22 cases, 43.14%), subsequently the rectum (15 cases, 29.41%), transverse colon (6 cases, 11.76%), sigmoid colon (5 cases, 9.8%), and the descending colon (3 cases, 5.88%). Fig 2 was revealed the tumor grades according to Dukes’ stage in colorectal cancer patients, with the most being stage B2 (50.98%) followed by stage C2 (41.18%) while stage D (7.84%) was the lowest prevalence stage among samples which have been enrolled in this study.

In patients with colorectal cancer, there was no significant relationship observed between the incidence of JCPyV, BKPyV, SV40, and HPVs viruses and gender (P value > 0.05) as indicated in Table 3. Based on the association between the age groups and presence of these viruses in CRC patients, no significant differences were found (Table 4).

**Table 3:** Classification of CRC patients (n = 51) based on gender and the type of human polyomaviruses or human papillomaviruses.

Virus type	Male	Female	*X* ^*2*^	*P*-value
	(n = 30)	(n = 21)		
SV40	9/30 (30%)	4/21 (19%)	0.78	0.377
BKPyV	7/30 (23.3%)	2/21 (9.5%)	1.621	0.203
JCPyV	0/30 (0%)	0/21 (0%)	-	-
HPVs	11/30 (36.7%)	11/21 (52.4%)	1.244	0.265

Chi-square statistical analyses used to determine P value significance range

**Table 4:** Prevalence of human polyomaviruses and human papillomaviruses regarding to age range in colorectal cancer patients.

	Human polyomavirus (HPyVs)			Human papillomavirus (HPVs)
	SV40	BKPyV	JCPyV	
Age (30–60)	6/21 (28.6%)	3/21 (14.3%)	0/21 (0%)	10/21 (47.6%)
Age (61–79)	7/30 (23.3%)	6/30 (20%)	0/30 (0%)	12/30 (40%)
*P*-value	0.673	0.598	-	0.589

The P value significance range is determined using chi-square statistical analyses.

Furthermore, our data revealed that 24/51 (47%) of CRC cases were positive for only one virus, while 10/51 (19.6%) of CRC cases were found to be co-infected with multiple virus types. The types and frequencies of virus co-infections in the CRC cohort were summarized in Table 5. A total of 9/51 (17.6%) cases were identified to have double infections, with BKPyV and HPVs being the most frequently observed co-infections (n = 4) among this group. Furthermore, 1/51 (2%) of the cases had triple infections, which included the three viruses SV40, BKPyV, and HPVs. The linear regression analysis of human papillomavirus and human polyomaviruses co-infections was significantly in SV40, BKPyV, and HPVs with *P* values < 0.001 (Table 6), demonstrating that the co-presence of more than one viral infection may be regarded as a high-risk factor for CRC incidence and could be strongly correlated with its occurrence.

**Table 5:** Categorizing a number of human polyomaviruses (HPyVs) co-infections and the most common combinations that affect patients with CRC.

Co-infections of HPyVs and HPVs (n = 10)	CRC Cases
*Double-infections (n = 9) 17*.*6%*	
SV40 and BKPyV	3
SV40 and HPVs	2
BKPyV and HPVs	4
*Triple-infections (n = 1) 2%*	
SV40, BKPyV, and HPVs	1

Human Polyomaviruses (HPyVs); Simian virus (SV40); BK polyomavirus (BKPyV); JC polyomavirus (JCPyV); papillomaviruses (HPVs); Colorectal cancer (CRC); number sample (n).

**Table 6:** Logistic regression analysis for human papillomaviruses and human polyomaviruses co-infections.

Variable	Un-standardized Coefficients		Standardized Coefficients	*t-test*	Significant	95% Confidence Interval	
	B	Std. Error	Beta			Lower Bound	Upper Bound
SV40	0.321	0.073	0.353	4.403	**0.0001**	0.175	0.468
BKPyV	0.672	0.078	0.672	8.613	**0.0001**	0.515	0.829
HPVs	0.245	0.064	0.305	3.854	**0.0001**	0.117	0.373

Dependent variable: co-infections

The relationship between the presence of viral co-infections and age, gender, tumor location, and Dukes’ stages in patients diagnosed with colorectal cancer was summarized in Table 7. The incidence of co-infection was in the development stage of CRC 70% in B2 stage and 30% in D stage. More importantly, our findings point out that human papilloma- and human polyoma- virus’s co-infections could perform an essential role in human CRC development, since it is obviously related with progressive tumor stage. The co-infections of human papillomavirus and human polyomaviruses were found to be strongly associated (p = 0.001) with advanced stages (B2 and D) of CRC, indicating that the co-incidence of HPyVs and HPVs can significantly worsen the prognosis of colorectal cancer, particularly in cases where the patient’s immune system is compromised, leading to increased susceptibility to opportunistic infections. Therefore, the presence of co-infections, particularly in advanced cases with lower immunity, could be considered a high-risk factor for the development and progression of colorectal cancer. In the final analysis, all the sequences obtained for the human papillomaviruses formed a distinct clade, which exhibited close clustering with previously isolated sequences of high-risk human papillomavirus type 16.

**Table 7:** Associated between the presence of co-infections with the demographic and pathological aspects in patients diagnosed with colorectal cancer.

Variable	Co-infections (+)		Co-infections (-)		*P* Value	Odds ratio (CI 95%)
	n (10)	%	n (41)	%		
Age at diagnosis						
Age ≤ 60	2	20%	19	46.3%		
Age > 60	8	80%	22	53.7%	0.122	3.45 (0.65–18.3)
Sex						
Male	7	70%	23	56.1%		
Female	3	30%	18	43.9%	0.334	0.55 (0.12–2.4)
Tumor location						
Ascending colon	2	20%	20	48.7%		
Descending colon	0	0%	3	7.3%		
Transverse colon	2	20%	4	9.8%		
Sigmoid colon	1	10%	4	9.8%		
Rectum	5	50%	10	24.4%	0.31	–
Dukes’ stage						
B2	7	70%	19	46.3%		
C2	0	0%	21	51.2%		
D	3	30%	1	2.5%	**0.001**	–

Number of colorectal cancer patients with positive (+) or negative (–) for virus co infections

Statistically significant values for p<0.05 using Fisher exact or Chi-square analyses

Odds ratio (OR) with a 95% confidence interval (CI); Dukes’ stages (B2 & C2 and D)

## Discussion

It is widely recognized that certain oncogenic viruses have the potential to contribute to the development of human cancers. Among these viruses, human papillomavirus (HPVs) and the four human polyomaviruses (SV40, BKPyV, JCPyV, and MCPyV) have been firmly established as being linked to human tumor formation [6–11]. However, the existing literature on the role of these viruses in the onset or progression of colon cancer is limited and contentious [8, 12]. Considering the scarcity of published data on the epidemiology of these human polyomaviruses and human papillomavirus in Egypt and across Africa, the objective of the current research was to focus on their molecular detection in colorectal cancer (CRC) patients. Remarkably, this study represents the first of its kind to uncover the presence of co-infections of these viruses in CRC tissue samples obtained from Egyptian populations.

Numerous research evaluated JCPyV presence, and/or sero-reactivity in CRC in order to explore the potential correlation between JCPyV and CRC. In general, these investigations found JCPyV footprints in both normal mucosal tissues and colorectal cancers. Contrary data were reported by other studies. Discordant results could be triggered, at least in part, by the use of various techniques and different markers in the study’s methodology [5]. In this investigation, tissue samples from our CRC patients and polyp’s subjects were evaluated for their incidence to JCPyV using PCR. Our data indicated that JCPyV was not detected in both human tissues from CRC patients and polyps samples. Consequently, an absence of JCPyV detection was shown by Ricciardiello *et al*. in an *in vitro* model of colonic cells [41].

On the other hand, Hassan *et al*. reported that 1.75% of patients with colorectal cancer tested positive for JCPyV DNA [12] which is in the same line with findings from El Hussein that 4.2% of patients examined positive for JCPyV DNA [42], as well as from Sarvari *et al*. who reported a low incidence of 1.42% JCPyV DNA in Iran [43]. Furthermore, the percentage was lower than that of Enam et al. who reported a remarkably high prevalence of 81% in colon cancers [44], and Rencic *et al*. who reported an 81.2% detection rate in colonic biopsy samples [45]. Although, JCPyV was detected in adjacent non-cancerous tissues as well as in normal tissues [46]. Fang *et al*. hypothesized that JCPyV infection may contribute to CRC progression [10]. The discordant in the results could be attributed, at least in part, to variations in techniques and gene targets used in the different studies. The use of different methodologies may lead to differences in sensitivity and specificity, potentially impacting the detection of JCPyV. Overall, the variability in results among these studies highlights the complexity of the relationship between JCPyV and CRC. Further research using standardized methodologies and larger sample sizes may help to clarify the role, if any, of JCPyV in the development and progression of colorectal cancer.

In relation to BKPyV infection, our study revealed the presence of BKPyV DNA in 19.6% of the CRC samples, which was not significantly different from the polyps’ group (21.1%). These findings are consistent with previous studies that have reported the presence of BKPyV proteins and DNA in colorectal adenocarcinoma tissues [47, 48]. Specifically, Casini et al. [47] identified BKPyV DNA in 27.7% (5/18) of CRC tissue samples, while Giuliani et al. [48] detected BKPyV DNA in 9% (6/66) of archival tumor samples. These studies provide further support for the presence of BKPyV in colorectal cancer and contribute to the growing body of evidence in this area. In contrast, previous reports found that BKPyV was not detected in colorectal cancer [12, 29, 49, 50]. These inconsistencies could be clarified by false-positive results brought on by contamination during experiments, the sensitivity of the techniques employed, the diversity of the population and sample size, and the prevalence of BKPyV in the examination area. The loss of BKPyV DNA provides another explanation [51–53]. Immune system selection against the dilution of episomal DNA may both result in the absence of viral DNA. Alternatively, the loss of BKPyV in human colorectal cancer tissues could be explained by a hit-and-run mechanism [54].

In the present study, SV40 was detected in 25.5% of our colorectal cancer specimens. Nevertheless, when compared with control group (10.5%) the results were not statistically significant. In agreement with our detection, SV40 can be detected in colon cancer tissue by a study in Italy and also detected SV40 without significant different from controls [55]. Additionally, another study was carried out in Sudan also detected the presence of SV40 with percent of 17% in CRC samples [12]. As a result, SV40 infection in various human populations across the globe has been documented by numerous organizations. In fact, normal tissues were found to have SV40 DNA sequences [32]. Conversely, in Khabaz *et al*. investigation, no SV40 DNA was found in any of the investigated colorectal tumors or control cases, suggesting that there is no correlation between SV40 and the incidence of colorectal adenocarcinoma [56]. There have been contradictory findings reported on the relationship between SV40 and various human tumors [32]. Hence, it is crucial to conduct further research on the involvement of polyomaviruses in colon cancer. While we examined the presence of DNA for JCPyV, BKPyV, and SV40 in both healthy and transformed tissues, it is worth noting that T-antigen expression could serve as a more valuable indicator of the association with cancer development. This limitation should be acknowledged in the current study and addressed in future investigations.

Our investigation revealed that 43.1% of CRC samples had positive human papillomavirus results. Since only a few countries in the Middle East and North Africa (MENA) have documented the presence of HPVs in CRC, data on the prevalence of HPV from MENA region are regrettably rare. There doesn’t seem to be agreement among these countries regarding the prevalence of HPVs, though; some are known to state a small percentage of HPV positive, while others report considerably higher rates [8]. The highest HPVs prevalence in CRC overall MENA region was detected in Syria (54%) [57] and Lebanon (64%) [58], that is slightly in line with our study’s results. Interestingly, data from Turkey indicates that 81% [59] and 82% [60] of CRC samples had HPVs infections, thus rendering it the country with the highest incidence of HPVs in CRC in the MENA region. Additionally, while there aren’t many findings linking HPVs to colorectal cancer in MENA countries, most of the evidence that is now accessible comes from Iran. It is interesting, nevertheless, that just one of the nine Iranian investigations revealed an elevated incidence of HPVs (83%) [61]. Most of the remaining studies illustrate a slightly lower prevalence, ranging from 0% to 23% [62–67]. In a comparable way, the majority of other MENA research demonstrated a decreased HPVs prevalence in colorectal cancer. For instance, only 15% [68] and 22% [69] of the CRC samples examined in two Egyptian investigations revealed an HPVs relationship. These studies’ data do not support the more widespread prevalence of HPVs (43.1%) that we observed in our investigation. These variations may be attributed to the different sample sizes and the technique sensitivity used to detect HPVs.

Concerning to the relationship of human polyomaviruses (JCPyV, BKPyV, and SV40) and human papillomavirus (HPVs) with gender and age range, the results of the current investigation indicated that the incidence of the above mentioned human polyoma- and human papilloma- viruses in colorectal cancer patients was not significantly correlated with either gender or age. Our finding was in the same line with previous study which indicated that these three polyomaviruses were not linked with age or gender [12]. In contrast, a study revealed that BKPyV and SV40 are more common in tumor patients, particularly males being more susceptible [70]. Finally, our examination displayed the feasibility of PCR assay for detection the co-infection of HPVs and HPyVs in colorectal cancer tissues. This is, as far as we know, the first investigation to identify co-infection between HPVs, BKPyV, and SV40 in Egyptian patients with CRC. The current results assessed these viral co-infections (19.6%) among colorectal cancer patients while failed to detect any co-infection in controls (0%). Especially, the incidence of co-infection was in the development stage of CRC 70% in B2 stage and 30% in D stage. The findings should require more extensive wider surveillance to completely clarify the epidemiology and actual status of these viruses’ co-presence in our country. These results were inconsistent with previous report that reveled, the prevalence of co-infection with multiple HPVs subtypes is notably high (50.8%) among CRC cases [8]. Furthermore, our findings suggest a potential association between human papilloma- and human polyoma- virus co-infections and human colorectal cancer (CRC) development. It is worth noting that this association appears to be linked to tumor stage progression, indicating a possible correlation. However, further studies are needed to fully understand the significance and mechanisms underlying this relationship.

## Conclusion

The prevalence of BKPyV, SV40, and HPVs co-infections in Egypt were determined to detect the DNA of these viruses in tissue samples from patients with colorectal cancer. These data might be utilized for epidemiological and diagnostic research in Egypt. In addition to their association with tumor progression, their co-infection was linked with advanced tumor stage. Since there is little information currently available regarding their human viral infection and co-infection in Egypt, these findings will be helpful for future research. Nonetheless, we agree that more research with a greater cohort is necessary to validate our findings. In this perspective, it is necessary to have a higher scientific investment in oncogenic viruses‑colorectal cancer relation, seeing that the identification of these viruses in colonic or rectal tissues can change the patient’s prevention, prognosis, and treatment.

## Materials and methods

### Participant selection and ethics

A total of 51 paraffin-embedded tissue blocks containing colorectal cancer samples (from 2019 to 2022), which had been previously diagnosed and stored in the archives of the Pathology Department at the Sednawi Hospital and Ahmed Maher Teaching Hospitals in Egypt, were included in this study. Furthermore, 19 polyp samples were collected for analysis. Clinicopathological data including age, gender, malignancy type, anatomical site, and modified Dukes’ grade were documented. The modified Dukes’ grade encompassed the following stages: Dukes A, indicating invasion into the bowel wall without penetration; Dukes B, signifying invasion through the bowel wall, penetrating the muscle layer but without lymph node involvement; Dukes C, denoting the presence of lymph node involvement; and Dukes D, representing widespread metastases. The control samples were collected from individuals who did not have cancer (polyps) and included surrounding normal tissue and distant surgical margins. For the purposes of this investigation, every tissue block was serially sectioned. The Research Ethics Committee of Faculty of Medicine in Beni-Suef University, Egypt provided the study ethical approval (No: FMBSUREC/09072023/Shaaban). The consent was not obtained because the data were analyzed anonymously and the study adhered to the principles outlined in the Second Declaration of Helsinki.

### Extraction of DNA

The QIAamp DNA FFPE Kit (Qiagen, Hilden, Germany) was used to extract genomic DNA from the paraffin-embedded tissue samples in accordance to the manufacturer’s instructions. Until using it again, the extracted DNA was kept at -80°C after it had been eluted in 50 μL of elution buffer. The isolated DNA’s concentrations were analyzed via Nano Drop spectrophotometer (ThermoFisher Scientific).

### Real time PCR for polyomaviruses

For the detection of SV40, BKPyV, JCPyV Polyomaviruses; the DNA amplifications were performed using a Rotor Gene Q Platform (Qiagen, Germany) following the manufacturer’s guidelines. β- globin was utilized as an internal control. PCR reactions were prepared with a volume of 50 μl. The reaction mixture consisted of 900 nM of each primer, 100 nM of TaqMan FAM-MGB probe, and 25 μl of the 2× TaqMan Universal PCR Master Mix. For each reaction, 10 μl of either the positive controls (pBKV (34–2) plasmid; ATCC & positive samples) or the test DNA samples was added in duplicate. The amplification of all target genes followed the same reaction conditions. The thermal cycling protocol included an initial step of incubation at 50°C for 2 minutes, followed by denaturation at 95°C for 10 minutes. This was followed by 40 cycles of denaturation at 95°C for 15 seconds, followed by annealing and extension at 60°C for 1 minute. During the PCR process, the amplification data, which reflects the increase in reporter fluorescence, were continuously monitored in real-time [39]. To determine the threshold, it was set at 10 standard deviations above the mean background fluorescence observed in the initial 15 cycles. Notably, the CT values of the standard dilutions displayed an average separation of approximately three cycles. This observation is consistent with the expected doubling of PCR product quantity with each cycle, indicating a roughly 10-fold increase requiring approximately 3.0 cycles. For detailed information on the sequences of the primers and probes utilized, please refer to Table 8.

**Table 8:** Primer and probes sequences used in the present study.

Virus	Gene	Primer	Sequence	Reference
β‐globin		Forward	5ʹ-TGCACGTGGATCCTGAGAACT-3ʹ	[38]
		Reverse	5ʹ-AATTCTTTGCCAAAGTGATGGG-3ʹ	
		Probe	5ʹ-CAGCACGTTGCCCAGGAGCCTG-3ʹ	
BKPyV	LT ag	Forward	5ʹ-TTGACTAAGAAACTGGTGTAGATC-3ʹ	[39]
		Reverse	5ʹ-AGAGTGGGAGTCCTGGTGGAGTTCC-3ʹ	
		Probe	5-’AATCTTCATCCCATTTTTCA-3’	
JCPyV	LT ag	Forward	5ʹ-GAGTGTTGGGATCCTGTGTTTTC-3ʹ	[39]
		Reverse	5ʹ-GAGAAGTGGGATGAAGACCTGTTT-3ʹ	
		Probe	5-GTTGGGATCCTGTGTTTTCAT-3’	
SV40	LT ag	Forward	5ʹ-TTAGCAATTCTGAAG GAAAGTCCTTG-3ʹ	[39]
		Reverse	5ʹ- ACCTGTTTTGCTCAGAAG-3ʹ	
		Probe	5’- ATGTTGAGAGTCAGCAGTAGCC-3	
HPVs	MY09	Forward	5ʹ-CGTCCMARRGGWACTGATC-3ʹ	[40]
	MY11	Reverse	5ʹ-GCMCAGGWCAGGWCATAAYAATGG-3ʹ	

### Real time PCR for human papillomaviruses

The amplification was performed using the Hybribio Limited Kit including positive and negative controls to detect 13 High-risk HPVs Real-time TaqMan PCR Kit (HBRT-H13; Bonham Trade Centre, Bonham Strand Sheung Wan, HONG KONG) following the manufacturer’s instructions. The kit was specifically designed for DNA purification to detect 13 HPVs types includes HPVs 16, 18, 31, 33, 35, 39, 45, 51, 52, 56, 58, 59, and 68. The eluted DNA was subjected to amplification that consisted of an initial denaturation step at 95°C for 10 minutes, followed by 45 cycles of denaturation at 95°C for 30 sec, annealing at 60°C for 50 sec, and extension at 72°C for 20 sec.

### PCR & sequencing for genotyping of human papillomaviruses

Samples that tested positive for HPVs underwent genotyping using the QIAGEN PCR kit, following the methodology outlined by Nishiwaki et al. [11]. The DNA amplification process involved an initial denaturation step at 95°C for 15 minutes, followed by 40 cycles of denaturation at 94°C for 30 seconds, annealing at 65°C for 90 seconds, and extension at 72°C for 90 seconds. A final extension was performed at 72°C for 10 minutes. Subsequently, the amplicons were electrophoreses in a 2% agarose gel, stained with ethidium bromide, and visualized under UV light. Positive amplicons were purified using the MEGA total fragment DNA purification kit (iNtRON Biotechnology, Hong Kong, China). These purified amplicons were then sent to Colors Laboratory, Egypt, for sequencing. The obtained sequences were aligned and compared to the HPVs sequences available in the NCBI Gene Bank.

### Statistical analysis

The results were analyzed via SPSS version 22. The quantitative variables were presented as mean ± SD using ANOVA test. The relationships between categorical variables which presented as number (%) were determined using the Chi-square or Fisher exact analyses. A linear regression test was used to find out the association of viral co-infections. *P*-values < 0.05 were revealed statistically significant.
